# Supplementary material for: Effect of myofibre architecture on ventricular pump function by using a neonatal porcine heart model: from DT-MRI to rule-based methods
Source: R Soc Open Sci. 2020 Apr 8;7(4):191655. doi: 10.1098/rsos.191655 (PMC7211874; doi:10.1098/rsos.191655)
Supplement: Details of co-registration of LDDMM, active contraction model, parameter sensitivity study and summary of literature-based fibre rotation angle [file rsos191655supp1.pdf]

# Supplementary Material

## S.1 Co-register two bi-ventricular geometries using *Deformetrica*

To warp a template ($C_\alpha$: the canine bi-ventricle) to a target ($C_\beta$: the neonatal porcine heart), we minimize the loss function

$$f(\mathbf{q}, \boldsymbol{\mu}) = d(\Phi_{\mathbf{q},\boldsymbol{\mu}}(C_\alpha), C_\beta)^2 + R(\mathbf{q}, \boldsymbol{\mu}) \tag{A1}$$

where the first term measures the distance between the template and target, i.e. how well the deformed template shape is close to the target shape, and the second term acts as a regularizer. $\Phi_{\mathbf{q},\boldsymbol{\mu}}$ is a diffeomorphism mapping, which is fully parameterized by the initial control points $\mathbf{q}$ and the momenta $\boldsymbol{\mu}$, the evolution equations for $\mathbf{q}$ and $\boldsymbol{\mu}$ follow the "Hamiltonian" system. For a shape represented by a triangulated surface with $N_e$ linear triangles, the centres $(\mathbf{c}_p)_{p=1,\ldots,N_e}$ and the normals $(\mathbf{n}_p)_{p=1,\ldots,N_e}$ of all triangles can be readily calculated. The distance between the two triangulated surfaces ($C_\alpha$ and $C_\beta$) is then given by the varifold distance[1], by ignoring normal orientations,

$$\begin{aligned} d(C_\alpha, C_\beta)^2 &= d((\mathbf{n}_p^\alpha, \mathbf{c}_p^\alpha)_{p=1,\ldots,N_e^\alpha},\, (\mathbf{n}_q^\beta, \mathbf{c}_q^\beta)_{q=1,\ldots,N_e^\beta})^2 \\ &= \sum_p \sum_q K\left(\mathbf{c}_p^\alpha, \mathbf{c}_q^\beta\right) \frac{((\mathbf{n}_p^\alpha)^T\, \mathbf{n}_q^\beta)^2}{|\mathbf{n}_p^\alpha||\mathbf{n}_q^\beta|} \end{aligned} \tag{A2}$$

where $K(\mathbf{c}_p, \mathbf{c}_q) = \exp(-|\mathbf{c}_p - \mathbf{c}_q|^2/\sigma^2)$ is a Gaussian kernel with width $\sigma$. Eq. (A1) is optimized with the steepest gradient decent or the L-BFGS method implemented in the *Deformetrica* package with respect to $\boldsymbol{\mu}$ to determine a diffeomorphism mapping $\Phi$. Details of the LDDMM framework can be found in[2].

## S.2 Active contraction model

The full description of active contraction is as following

$$T_a(t,l) = \frac{T_{\max}}{2} \frac{\mathrm{Ca}_0^2}{\mathrm{Ca}_0^2 + \mathrm{ECa}_{50}^2\,(l)} (1 - \cos(\omega(t,l))) \tag{A3}$$

where

$$\mathrm{ECa}_{50}(l) = \frac{\mathrm{Ca}_{0\max}}{\sqrt{e^{B(l-l_0)} - 1}}$$

$$\omega(t,l) = \begin{cases} \pi \frac{t}{t_0} & \text{for} \quad 0 \leqslant t \leqslant t_0 \\ \pi \frac{t - t_0 + t_r(l)}{t_r} & \text{for} \quad t_0 < t \leqslant t_0 + t_r \\ 0 & \text{for} \quad t > t_0 + t_r \end{cases}$$

$$t_r(l) = ml + b$$

$$l = l_r \sqrt{2E_{\mathrm{ff}} + 1}$$

where $T_{\max}$ is the isometric tension at the largest sarcomere length and highest calcium concentration, $\mathrm{Ca}_0$ is the peak intracellular calcium concentration; $m$ and $b$ are constants that govern the shape of the linear relaxation duration and sarcomere length relaxation; $E_{\mathrm{ff}}$ is the Lagrange strain in the fibre direction; $B$ is a constant that governs the shape of the peak isometric tension-sarcomere length relation; $l_r$ is the sarcomere length with the stress-free condition whilst $l_0$ is the sarcomere length that does not produce active stress[3]. Active parameters values[4] can be find in Table A1

Table A1: Parameter values for the time varying elastance active tension constitutive model.

| | $t_0$ (ms) | $m$ (s $\mu$m$^{-1}$) | $b$ (s) | $l_0$ ($\mu$ m) | $B$ ($\mu$m$^{-1}$) | $\mathrm{Ca}_0$ ($\mu$M) | $\mathrm{Ca}_{0\max}$ ($\mu$M) | $T_{\max}$ (kPa) | $l_r$ ($\mu$m) |
|---|---|---|---|---|---|---|---|---|---|
| LV | 150 | 1048.9 | -1.7 | 1.58 | 4.750 | 4.35 | 4.35 | 180 | 1.85 |
| RV | 150 | 1048.9 | -1.7 | 1.58 | 4.750 | 4.35 | 4.35 | 135 | 1.85 |

## S.3 Parameter sensitivity and inference study of myocardial property

A sensitivity study is performed with case LDDMM. The so-called "one-point" approach is employed here by varying one parameter at a time and others kept same. Parameters ($a$, $b$, $a_{\mathrm{f}}$, $b_{\mathrm{f}}$, $a_{\mathrm{n}}$, $b_{\mathrm{n}}$, $a_{\mathrm{fs}}$, $b_{\mathrm{fs}}$, $a_{\mathrm{fn}}$, $b_{\mathrm{fn}}$ and $T_{\max}$) are first doubled and then halved from the values in Table 3 (the main text). Figure A1(a,b) shows the normalized end-diastolic and end-systolic volumes with respect to case LDDMM. It can be found that end-diastolic volume is mostly affected by $a$, $b$, $a_{\mathrm{f}}$ and $a_{\mathrm{n}}$, while end-systolic volume is mostly affected by $T_{\max}$. Figure A1 (c) further shows the changes of ejection fractions. Both LV and RV ejection fractions are reduced when doubling $a$, $b$, $a_{\mathrm{f}}$, $a_{\mathrm{n}}$, and vice versa. $T_{\max}$ has the largest effect on LV and RV ejection fractions, while other parameters have little influences.

As mentioned previously, it is not our intention to develop a personalized bi-ventricular model for this neonatal porcine heart, instead we seek to construct a general model with physiologically-correct pump function, i.e. eject fraction > 50%. Currently, there is no consistent way on how to adjust parameters derived from ex-vivo experiments to in vivo models. Published studies have suggested passive parameters estimated from ex vivo experiments can over-estimate the stiffness in vivo[5,6,7]. Thus in many studies, parameters of in vivo myocardium are scaled from ex vivo experiments[8,6] in order to match in vivo measurements. The similar approach is followed in this study by scaling $a$, $a_{\mathrm{f}}$, $a_{\mathrm{n}}$, $a_{\mathrm{fs}}$, $a_{\mathrm{fn}}$ to match targeted end-diastolic volumes. In this study, the end-diastolic volume for the LV is $(V_0 + 1)$ mL, and $(V_0 + 0.6)$ mL for the RV with $V_0$ being the reference volume of the LV or RV, respectively. $T_{\max}$ is then determined by achieving targeted ejection fractions. Figure A2 illustrates how the mismatch between the targeted value and the predicted value is reduced by the passive scaling factor and $T_{\max}$ during the inference procedure for the LV as an example. Figure A2(a) is the mismatch of LV end-diastolic volume with respect to the scaling factor, which is defined as $|(\mathrm{EDV}^{\mathrm{predict}} - \mathrm{EDV}^{\mathrm{target}})/\mathrm{EDV}^{\mathrm{target}}|$, and figure A2(b) is the mismatch of LV ejection fraction ($|(\mathrm{EF}^{\mathrm{predict}} - \mathrm{EF}^{\mathrm{target}})/\mathrm{EF}^{\mathrm{target}}|$) with respect to $T_{\max}$. From figure A2, it can be seen that both the passive scaling factor and myocardial contractility ($T_{\max}$) can be nicely

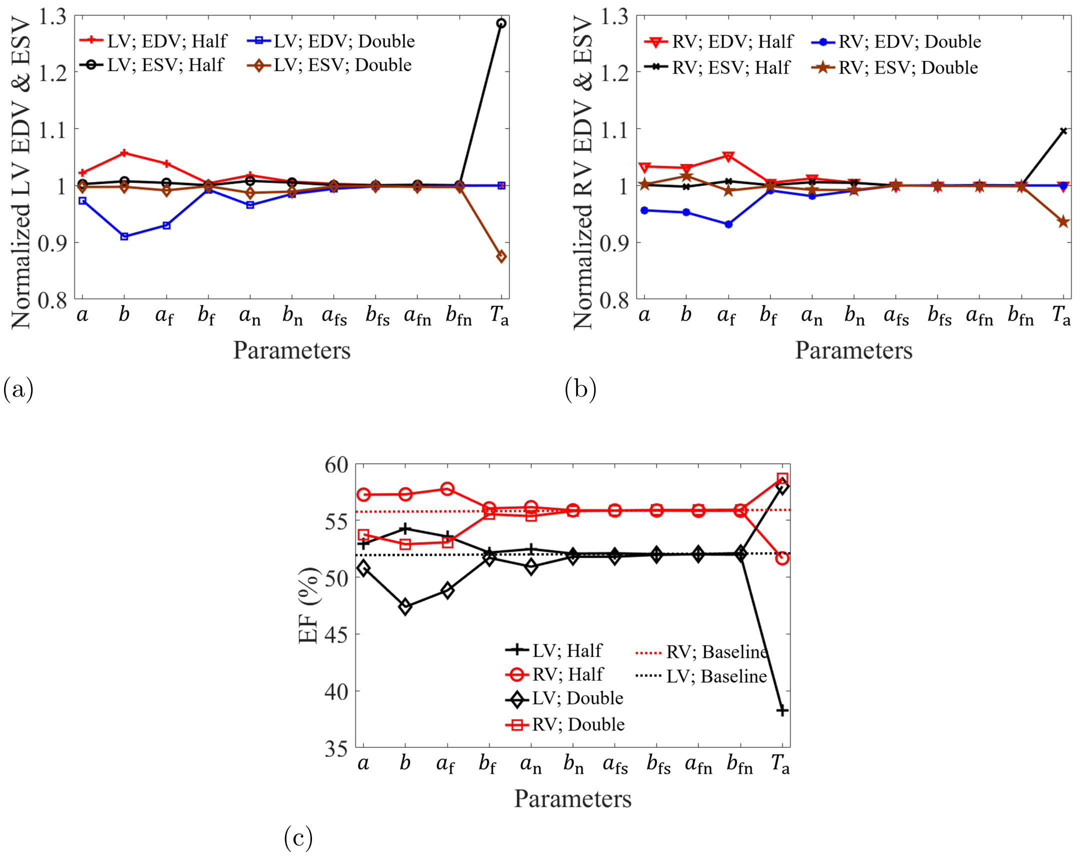


Figure A1: Myocardial material parameter sensitivity study, including $a$, $b$, $a_\mathrm{f}$, $b_\mathrm{f}$, $a_\mathrm{n}$, $b_\mathrm{n}$, $a_\mathrm{fs}$, $b_\mathrm{fs}$, $a_\mathrm{fn}$, $b_\mathrm{fn}$) and the active parameter ($T_\mathrm{max}$). (a) normalized EDV and ESV values of the LV and (b) the RV with respect to the corresponding baseline values; (c) EF values. The baseline values of LV and RV are from the simulation with parameter values in Table 3 (the main text).

determined by matching targeted values. Note that this approach will only provide one set of possible parameters, to uniquely infer each parameter of myocardial material property can be extremely challenging due to various difficulties[6], i.e. limited measured data, parameter correlation, etc.

## S.4 Myofibre rotation angles from literature

Table A2 is the summary of myofibre rotation angles from published experimental and modelling studies.

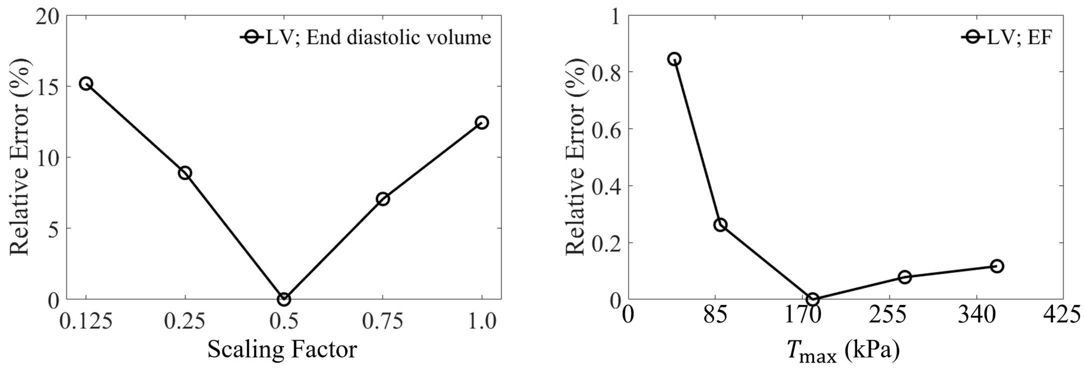


(a) (b)

Figure A2: Relative errors in EDV (a) and EF (b) of the LV when inferring reasonable model parameters by matching targeted end-diastolic volume $(V_0 + 1)$ mL with $V_0$ the initial value and ejection fraction 52%.

Table A2: Summary of fibre rotation angles from published experimental modelling studies.

| Experimental studies | | | Modelling studies | | |
|---|---|---|---|---|---|
| | Epicardium | Endocardium | | Epicardium | Endocardium |
| Anterior LVFW[9] | $-51.1 \pm 3.8^o$ | $51.1 \pm 3.8^o$ | Doste et al[10] | $-60.0^o$ | $60.0^o$ |
| Anterior RVFW[9] | $-70.5 \pm 6.5^o$ | $70.5 \pm 6.5^o$ | Sack et al[11] | $-60.0^o$ | $60.0^o$ |
| Posterior LVFW[9] | $-40.2 \pm 2.9^o$ | $40.2 \pm 2.9^o$ | Bayer et al[12] | $-50^o$ | $40^o$ |
| Posterior RVFW[9] | $-22.1 \pm 6.2^o$ | $22.1 \pm 6.2^o$ | Wang et al[5] | $-60^o$ | $60^o$ |
| Excluding apex[8] | $-37. \pm 22.44^o$ | $66.5 \pm 16.6^o$ | Gao et al[6] | $-60^o$ | $60^o$ |
| Anterior[13] | $-35.7 \pm 8.6^o$ | $38.5 \pm 6.1^o$ | Barbarotta et al[14] | $-60^o$ | $60^o$ |
| Lateral[13] | $-41.0 \pm 8.0^o$ | $29.7 \pm 6.6^o$ | | | |
| Posterior[13] | $-57.0 \pm 6.2^o$ | $39.5 \pm 10.6^o$ | | | |

# References


1. Charon N, Trouvé A. 2013 The varifold representation of nonoriented shapes for diffeomorphic registration. *SIAM Journal on Imaging Sciences* **6**, 2547–2580.

2. Durrleman S, Prastawa M, Charon N, Korenberg JR, Joshi S, Gerig G, Trouvé A. 2014 Morphometry of anatomical shape complexes with dense deformations and sparse parameters. *NeuroImage* **101**, 35–49.

3. Guccione J, McCulloch A. 1993 Mechanics of active contraction in cardiac muscle: part I—constitutive relations for fiber stress that describe deactivation. *Journal of biomechanical engineering* **115**, 72–81.

4. Sack KL, Davies NH, Guccione JM, Franz T. 2016 Personalised computational cardiology: Patient-specific modelling in cardiac mechanics and biomaterial injection therapies for myocardial infarction. *Heart failure reviews* **21**, 815–826.

5. Wang H, Gao H, Luo X, Berry C, Griffith B, Ogden R, Wang T. 2013 Structure-based finite strain modelling of the human left ventricle in diastole. *International journal for numerical methods in biomedical engineering* **29**, 83–103.

6. Gao H, Li W, Cai L, Berry C, Luo X. 2015 Parameter estimation in a Holzapfel–Ogden law for healthy myocardium. *Journal of engineering mathematics* **95**, 231–248.

7. Genet M, Lee LC, Nguyen R, Haraldsson H, Acevedo-Bolton G, Zhang Z, Ge L, Ordovas K, Kozerke S, Guccione JM. 2014 Distribution of normal human left ventricular myofiber stress at end diastole and end systole: a target for in silico design of heart failure treatments. *Journal of applied physiology* **117**, 142–152.

8. Sack KL, Aliotta E, Ennis DB, Choy JS, Kassab GS, Guccione JM, Franz T. 2018 Construction and validation of subject-specific biventricular finite-element models of healthy and failing swine hearts from high-resolution DT-MRI. *Frontiers in physiology* **9**, 539.

9. Ahmad F, Soe S, White N, Johnston R, Khan I, Liao J, Jones M, Prabhu R, Maconochie I, Theobald P. 2018 Region-Specific Microstructure in the Neonatal Ventricles of a Porcine Model. *Annals of biomedical engineering* **46**, 2162–2176.

10. Doste R, Soto-Iglesias D, Bernardino G, Alcaine A, Sebastian R, Giffard-Roisin S, Sermesant M, Berruezo A, Sanchez-Quintana D, Camara O. 2018 A rule-based method to model myocardial fiber orientation in cardiac biventricular geometries with outflow tracts. *arXiv preprint arXiv:1809.08297*.

11. Sack KL, Dabiri Y, Franz T, Solomon SD, Burkhoff D, Guccione JM. 2018 Investigating the role of interventricular interdependence in development of right heart dysfunction during LVAD support: A patient-specific methods-based approach. *Frontiers in physiology* **9**.

12. Bayer JD, Blake RC, Plank G, Trayanova NA. 2012 A novel rule-based algorithm for assigning myocardial fiber orientation to computational heart models. *Annals of biomedical engineering* **40**, 2243–2254.

13. Helm PA, Tseng HJ, Younes L, McVeigh ER, Winslow RL. 2005 Ex vivo 3D diffusion tensor imaging and quantification of cardiac laminar structure. *Magnetic resonance in medicine* **54**, 850–859.

14. Barbarotta L, Rossi S, Dedè L, Quarteroni A. 2018 A transmurally heterogeneous orthotropic activation model for ventricular contraction and its numerical validation. *International Journal for Numerical Methods in Biomedical Engineering* **34**, e3137.
